# Reproducibility and variability of left ventricular 4D flow in healthy volunteers

**DOI:** 10.1186/1532-429X-17-S1-P7

**Published:** 2015-02-03

**Authors:** Victoria Stoll, Aaron T Hess, Malenka M Bissell, Jonatan Eriksson, Petter Dyverfeldt, Tino Ebbers, Saul G Myerson, Carl Johan Carlhall, Stefan Neubauer

**Affiliations:** 1 University of Oxford Centre for Clinical Magnetic Resonance Research (OCMR), Division of Cardiovascular Medicine, Radcliffe Department of Medicine, Oxford, UK; 2 Center for Medical Image Science and Visualization (CMIV), Linköping University, Linköping, Sweden

## Background

Blood flow through the heart is a fundamental aspect of the function of the cardiovascular system. Left ventricular intra-cardiac flow, as assessed by retrospectively gated 4D flow, can be divided into 4 functional components; direct flow (DF), delayed ejection flow (DEF), retained inflow (RI) and residual volume (RV). Additionally the kinetic energy of these flow components can be calculated throughout the cardiac cycle. Previous studies have demonstrated differences in the proportions and kinetic energy of flow components between healthy volunteers and patients with dilated cardiomyopathy.

This study aims to assess the inter-scan reproducibility and variability of the LV 4D flow components in healthy volunteers.

## Methods

15 participants were prospectively enrolled. 5 participants underwent consecutive 4D flow and anatomical data acquisitions within the same scanning session. The other 10 participants underwent 2 data acquisitions separated by an interval of between 2-8 weeks.

## Results

The 5 paired data sets, for reproducibility, were assessed by the mean difference and the standard deviation in the 4 flow components between scans; DF -1.6 ± 3.2% (p=0.3 paired t-test), DEF 2.1 ± 2.9% (p=0.2), RI -0.1 ± 1.2% (p=0.9) and RV -0.5 ± 1.2% (p=0.5). The mean differences in end diastolic kinetic energy were: DF 0.4 ± 1.2 µJ/ml (p=0.5), DEF 0.5 ± 0.6 µJ/ml (p=0.1), RI 0.8 ± 0.6 µJ/ml (p=0.05) and for RV 0.1 ± 0.3 µJ/ml (p=0.4).

**Figure 1:**
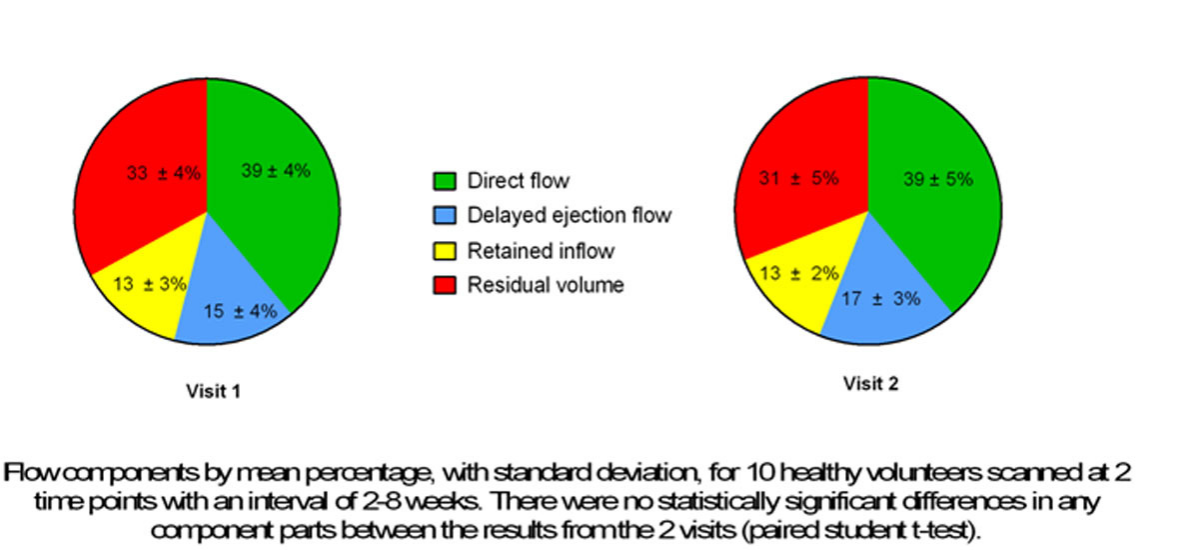
Volume flow components in relation to end diastolic volume

**Figure 2:**
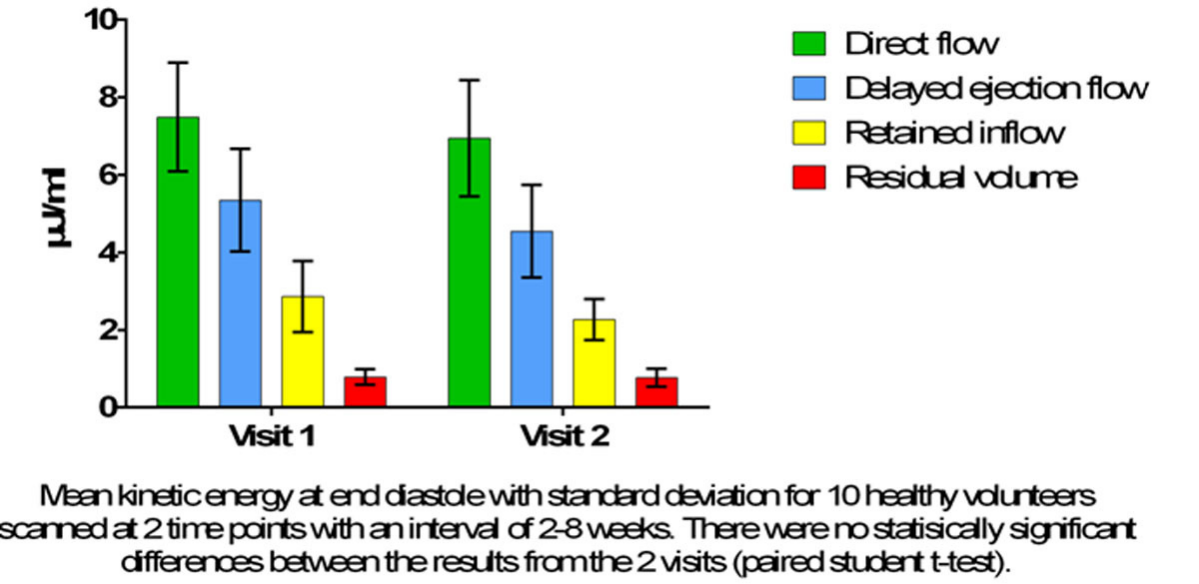
Kinetic energy at end diastole

The volume of the flow components in relation to the end-diastolic volume, for the 10 volunteers attending for 2 visits, are shown in figure 1. Mean differences for DF of 0.3 ± 3.8% (p=0.8), DEF 2.1 ± 3.7% (p=0.1), RI -0.002 ± 2.5% (p=0.9) and RV 1.8 ± 3.5% (p=0.1) were obtained. The mean kinetic energy at end diastole for each study visit is shown in figure 2.

## Conclusions

Left ventricular 4D flow data is reproducible, with a maximum standard deviation of 3% difference in the flow component percentages and less variability in the kinetic energy values at end diastole. For the data acquired with a time interval the largest individual change in flow component volumes was 8% suggesting a degree of physiological variability as well as variability due to the sequence and data analysis. This is the first study aiming to understand physiological variability over time of 4D flow data. Establishing physiological ranges will aid interpretation of data in patient groups in order to understand if changes are physiological or pathological.

## Funding

Dr Victoria Stoll is funded by the British Heart Foundation FS/12/14/29354. The research was supported by the National Institute for Health Research (NIHR), Oxford Biomedical Research Centre based at The Oxford University Hospitals Trust at the University of Oxford and The Swedish Heart and Lung Foundation.

